# Editorial: Explainable artificial intelligence for critical healthcare applications

**DOI:** 10.3389/frai.2023.1282800

**Published:** 2023-09-12

**Authors:** Zhe He, Rui Zhang, Gayo Diallo, Zhengxing Huang, Benjamin S. Glicksberg

**Affiliations:** ^1^School of Information, Florida State University, Tallahassee, FL, United States; ^2^Division of Computational Health Sciences, University of Minnesota, Minneapolis, MN, United States; ^3^Team AHeaD, Bordeaux Population Health INSERM U1219, University of Bordeaux, Bordeaux, France; ^4^College of Computer Science and Technology, Zhejiang University, Hangzhou, China; ^5^Hasso Plattner Institute for Digital Health, Icahn School of Medicine at Mount Sinai, New York, NY, United States

**Keywords:** explainable artificial intelligence, healthcare application, health informatics, electronic health records, medical image

## Introduction

With the recent advancements of Artificial Intelligence (AI), its applications in human health continue to be of focal interest. With the enormous amount of health-related data being generated and aggregated, especially from electronic health records (EHRs) and medical images, we are faced with an AI revolution that has been shown to accelerate clinical research, optimize healthcare quality, provide data-driven clinical decision support, and ultimately improve patients' health outcomes (Rajpurkar et al., 2022). Further, with the recent explosion of interest in generative large language model-based tools such as ChatGPT, AI is expected to further revolutionize healthcare delivery. However, there are also growing concerns that AI, with inadequate oversight and regulation, may advertently cause harm with its inherent biases, lack of transparency, and lack of interpretability, which are vital to garner its trustworthiness, especially in clinical practice. Although there are ethics-based AI principles and reporting guidelines such as Transparent Reporting of a multivariable prediction model of Individual Prognosis Or Diagnosis-AI (TRIPOD-AI) (Collins et al., 2021) and MINimum Information for Medical AI Reporting (MINIMAR) (Hernandez-Boussard et al., 2020), there isn't yet any guideline that covers transparency, fairness, explainability, and ethics at the same time. Recently, the US National Academy of Medicine is working with leading bioethics, research, and patient advocacy organizations to develop an AI Code of Conduct to support the equitable and responsible use of AI for health care and research. Similarly, as part of its digital strategy, the European Commission wants to regulate AI (the AI Act) to ensure better conditions for the development and use of this innovative technology (European Commission, 2023). Health and biomedical informatics are at the forefront of medical AI because informatics researchers study the optimal use of medical knowledge-driven techniques to benefit clinicians, clinical researchers, and patients, and without adequate buy-in, medical AI will not realize its intended value.

Why is explainability a major concern in medical AI and crucial for its trustworthiness? Traditional machine learning highly relies on laborious feature engineering, which may not be adequate for discovering complex patterns in high-dimensional EHRs or medical images, resulting in suboptimal performance. In contrast, deep learning (DL) methods enable machines to automatically detect the intricate relationships among features and extract meaningful knowledge from data. More recently, advanced DL models such as transformers and auto-encoders have shown promise in representing complex clinical data from multiple modalities, leading to significantly improvements in downstream tasks such as patient similarity identification, disease onset prediction, and deep phenotyping. However, DL algorithms are often perceived as black-box models because they incorporate high-degree interactions between input features through a multi-layer nonlinear structure with numerous neurons. To ensure the trustworthiness of DL models and justify their predictions in high-stakes healthcare applications, it is crucial for medical professionals to understand how the system generates specific predictions. Such explanations play an essential role in ensuring fairness and accountability in the clinical decision-making process. This aspect is important, as a single incorrect prediction from the system can have real-world consequences that may lead to serious medical errors (Obermeyer et al., 2019). Recognizing the significance of transparency, the European Union's General Data Protection Regulation (GDPR) was recently enacted to require organizations that use patient data for classifications and recommendations to provide on-demand explanations. Additionally, the White House has developed guidance on AI applications that emphasizes transparency as a fundamental principle for the stewardship of AI applications. Sufficient explanations of the AI models allow medical doctors to comprehend and trust AI-based clinical decision support systems. Consequently, research on explainable AI (XAI) in healthcare is increasing to address these concerns (Payrovnaziri et al., 2020).

To establish an open-access platform for researchers to disseminate novel methods and applications of XAI in critical healthcare applications, we dedicate this Research Topic to include original research articles that present innovative XAI methodologies and applications in healthcare that aim to ensure fairness, accountability, and trustworthiness of AI systems. Following a rigorous review, revision, and selection process, we finally accepted four articles including three original research articles and one review article.

## Published studies in this Research Topic

In Kiseleva et al., the authors from Belgium and France reviewed the legislation and policies regarding transparency in health AI models in the European Union and suggested that transparency is an umbrella concept that includes interpretability and explainability and it should be achieved through both technical and non-technical measures. The article also discusses the importance of making AI in healthcare externally transparent to patients such that patients should be informed and give informed consent to use it. They also stressed that the implementation of a transparency system must be contextualized.

In An et al., a team of researchers from the US addressed a critical problem in AI-based breast cancer screening, the lack of high-quality pixel-level annotated data for anomalous tissue by introducing LatentCADx, a DL segmentation model that can precisely annotate cancer lesions underlying hand-drawn annotations. This architecture is based on the ResNet convolution and allows weights learned from a ResNet model to be transferred to another ResNet model as well as selective training of layers. The performance of LatentCADx using a publicly available dataset of 2,620 mammogram case files reached 0.97 AUROC. In addition, qualitative evaluation of the segmentation showed great clarity and specificity with weakly-supervised segmentation, which ensures the interpretability of the model.

In Fujihara et al., a research team from Japan developed a ML model to predict changes in future weight in the subsequent 3 years using 67,021 Japanese individuals who had initial health examinations at the Niigata Association of Occupational Health. The predictors include body mass index, smoking and alcohol intake status, exercise habit, skipping breakfast, walking speed, eating speed, and other medical conditions such as history of dyslipidemia, diabetes, and hypertension. They used heterogeneous mixture learning technology (HMLT) which allows a mixture of data following different patterns. This technique uses predicted weights after 1 year to predict weights after 2 years, and then 3 years. The overall root mean square error (RMSE) was 1.914. For interpretability, HMLT can generate a decision-tree-like structure with five linear models based on rules that maximize the prediction accuracy. As expected, they found people with healthy lifestyle habits tended to have weight reduction, while people with unfavorable lifestyle habits tended to increase weight.

In Chu et al., researchers developed ML models to predict physical functions after older adults are discharged from hospitalization in Taiwan. In the experiments, 52 clinical features were considered when evaluating XGBoost, random forest, logistic regression models, which yield AUROC of 98%, 98%, and 97%, respectively. Using SHAP values for interpretability assessment, they found that for the XGBoost model, activity of daily living at baseline, activity of daily living at admission, and mini nutritional status during admission were the top three predictors.

To summarize, XAI is instrumental in improving potential buy-in among healthcare professionals. But XAI alone is not sufficient for the trustworthiness of AI, other factors including fairness of the model, transparency of the biases in the training data, external validity of the models, and clinical utility of the findings are all important for the equitable and responsible use of AI for healthcare. As AI models make their way into the clinic via Software as a Medical Device (SaMD) designation, we are looking forward to seeing authoritative ethical AI guidelines and more exciting research that pushes this research agenda forward.

## Author contributions

ZHe: Writing—original draft, Writing—review and editing. RZ: Writing—review and editing. GD: Writing—review and editing. ZHu: Writing—review and editing. BG: Writing—review and editing.
